# The Long-Term Impact of Fuel Exposure (LIFE) Study: A Tri-Service Cohort of United States Veterans with Military Occupational Exposure to Jet Fuels

**DOI:** 10.3390/ijerph22091337

**Published:** 2025-08-27

**Authors:** Elizabeth R. Heitz, Nicholas A. Tilton, Justin G. Bergeron, Gregory Wolff, Jennifer A. Rusiecki, Aaron I. Schneiderman, Warren S. Monks, Christopher Edwards, Gillon D. Marchetti, Terra D. Vincent-Hall

**Affiliations:** 1 Exposure Science Program, Health Outcomes Military Exposures, Veterans Health Administration, Department of Veterans Affairs, Washington, DC 20420, USA; 2 Epidemiology Consult Service, US Air Force School of Aerospace Medicine, Wright-Patterson AFB, Dayton, OH 45433, USA; 3 Department of Medicine, Uniformed Services University of the Health Sciences, Bethesda, MD 20814, USA; 4 Epidemiology Program, Health Outcomes Military Exposures, Veterans Health Administration, Department of Veterans Affairs, Washington, DC 20420, USA; 5 Directorate of Clinical Public Health and Epidemiology, Defense Centers for Public Health-Aberdeen, Aberdeen Proving Ground, Aberdeen, MD 21010, USA; 6 711th Human Performance Wing, US Air Force School of Aerospace Medicine, Wright-Patterson AFB, Dayton, OH 45433, USA; 7 Military Exposure Team, Compensation Service, Veterans Benefits Administration, Department of Veterans Affairs, Washington, DC 20420, USA

**Keywords:** Veterans, military, occupational health, exposure assessment, jet fuel, aircraft fuel, health outcomes, chronic disease, long-term effects, retrospective cohort

## Abstract

Jet fuels are a complex mixture of hydrocarbons and performance additives, including some compounds with established human toxicity. They represent a significant occupational hazard for military personnel; however, little is known about possible long-term health effects, particularly following cessation of exposure. In response to United States (US) Veterans’ concerns, the US Department of Veterans Affairs (VA) and the Department of Defense (DoD) launched a large retrospective cohort study to assess the impact of military occupational jet fuel exposure on Veterans’ health. The Long-Term Impact of Fuel Exposure (LIFE) cohort consists of over 1.3 million Veterans who entered service on or after 1 January 1995, including both individuals with jet fuel-exposed occupations in their service history and a random sample of unexposed Veterans. Data from multiple VA and DoD administrative datasets were linked to evaluate morbidity, disability, and mortality endpoints. Analyses are underway to assess associations between jet fuel exposure and adverse health outcomes in multiple body systems. This study represents the largest effort to date to investigate these effects, with the intention of informing policies affecting Veterans for years to come.

## 1. Introduction

Jet fuels are a common occupational hazard among military service members throughout the industrialized world [1,2]. The United States (US) military uses a significant amount of jet fuel daily. US service members are exposed in expected settings, such as during aircraft refueling and maintenance [2,3,4]. Exposure also occurs in settings unrelated to aviation, since jet fuels are used as a power source for military ground transport vehicles and equipment. It is used as a cleaning and decontaminating agent and has previously been used as an accelerant in burn pit fires [5,6].

In the 1990s, the US military adopted a “single fuel for the battlefield” concept to simplify fueling logistics in operational settings [7]. Since then, both the Army and Air Force (USAF) have primarily relied on JP-8, while the USAF also uses Jet A, a commercial fuel with a similar composition. The Navy utilizes JP-8 for land-based operations and JP-5 for shipboard activities due to its higher flash point, which enhances safety on aircraft carriers. JP-8 is also the primary jet fuel used by other North Atlantic Treaty Organization member countries [8]. These fuels are kerosene-based, with performance additives that improve their thermal stability, anti-icing properties, corrosion resistance, and static dissipation.

Exposure to jet fuel has previously been associated with acute health effects, including respiratory, neurological, and cognitive effects [8,9,10,11,12,13,14,15,16]. These effects are consistent with the acute effects of kerosene exposure, which constitutes approximately 98% of jet fuel by volume [4,5,6]. Symptoms of kerosene exposure vary by route: aspiration may lead to rapid or labored breathing, cough, restlessness, and lethargy or unconsciousness [6,17,18,19], while dermal exposure can cause itching, burning, and blistered skin [4,20,21,22]. Some of the minor components of jet fuel have also been identified as toxic to humans. For example, xylenes (<1% of jet fuel by volume) are known to cause both acute ocular irritation and long-term neurological effects [23,24,25]; toluene (<1% of jet fuel by volume) has been linked to nervous system and cognitive dysfunction [26,27,28]; and benzene (<1% of jet fuel by volume) is a known human carcinogen [29,30].

Still, the health effects of these chemicals as a mixture, and importantly their impact on long-term health outcomes, are not well understood. The Department of Veterans Affairs (VA) conducted a systematic literature review of outcomes associated with occupational jet fuel exposure to assess the available evidence and guide future research [31]. The review identified relevant studies from North America, Europe, Australia, and Asia. Together these studies provided limited evidence of associations between jet fuel exposure and neurological, cognitive, behavioral, respiratory, and neoplastic outcomes. However, the evidence regarding effects on other health outcomes was deemed inconclusive. None of the 28 studies reviewed assessed health effects according to exposure duration, and few provided adequate follow-up to examine long-term health outcomes, particularly those that developed after cessation of exposure. Studies also lacked data on health outcomes in females, as well as important confounders including race and ethnicity, lifestyle risk factors (e.g., smoking, alcohol consumption), and co-morbidities.

To begin to address these knowledge gaps, VA is collaborating with the USAF School of Aerospace Medicine, the Uniformed Services University of the Health Sciences, and the Defense Centers for Public Health–Aberdeen on the Long-term Impact of Fuel Exposure (LIFE) Study. As a retrospective cohort study, the LIFE Study will examine chronic health outcomes potentially associated with service-related jet fuel exposure in a large cohort of Veterans. The LIFE Study’s findings will inform the healthcare of millions of current and future Veterans impacted by jet fuel exposure, while its methods will establish a collaborative approach to addressing emerging exposure issues.

### Specific Aims

Three specific aims were defined for the LIFE Study: (1) To determine whether occupational exposure to jet fuel during military service is linked to an increased risk of long-term adverse health outcomes among Veterans. (2) To assess the association between military occupational exposure to jet fuel and the risk of mortality in Veterans. (3) To evaluate the relationship between occupational jet fuel exposure and the likelihood of filing a claim and receiving disability compensation for chronic health conditions among Veterans. Analyses addressing each aim will also consider whether observed associations are influenced by personal demographic characteristics (such as age, sex, and race/ethnicity), military service factors (including deployment history), and by the duration, intensity, and cumulative level of jet fuel exposure.

## 2. Materials and Methods

### 2.1. Overview

**Figure 1:**
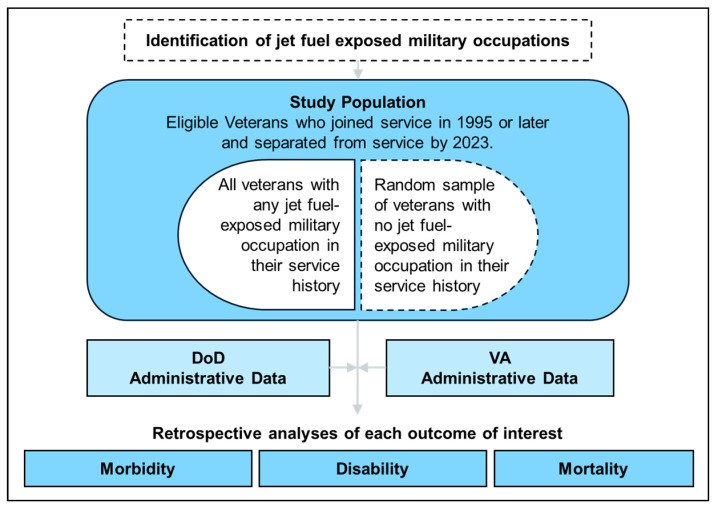
Flow diagram overview of LIFE Study procedures.

This study utilizes a retrospective cohort design with occupational jet fuel exposure primarily defined using standardized military occupational codes. All eligible Veterans with probable exposure, based on their occupational history, and a sample of unexposed Veterans are included in the cohort. Comprehensive military service history, health encounters, disability claims, and mortality data will be collected from VA and Department of Defense (DoD) administrative data sources and linked using unique identifiers. These data will be used to assess various health outcomes at multiple endpoints (i.e., morbidity, disability, and mortality) to develop a comprehensive assessment of the health effects of occupational fuel exposure on Veterans. Analyses will be prioritized based on the concerns expressed by Veterans and other stakeholders and evidence of association identified in existing research. An illustration of the LIFE Study’s approach is shown in Figure 1.

### 2.2. Study Population

The LIFE Study cohort comprises Veterans who joined the Army, USAF, Navy, or Marine Corps on or after 1 January 1995 and separated from service by 31 December 2023, a population whose jet fuel exposures were primarily to JP-8 and Jet A or JP-5. To ensure that all members of the cohort completed their military occupational training and their first service contracts, only Veterans who served in active duty for at least two years and whose longest held rank was Enlisted-2 (E2) or higher are included. Veterans who served in the National Guard or Reserves are excluded due to the possibility that they may have also encountered additional unmeasured occupational jet fuel exposures in civilian careers. Veterans who served in multiple services are excluded as exposure may differ across branches of service, even within similar occupations.

The LIFE Study cohort includes all Veterans who met these inclusion criteria and worked in any jet fuel-exposed occupation during their service, along with a reference group of Veterans with no history of jet fuel-exposed occupations who otherwise met the inclusion criteria. The unexposed sample was selected at random at a targeted one-to-one ratio with the exposed population.

### 2.3. Data Sources

**Figure 2:**
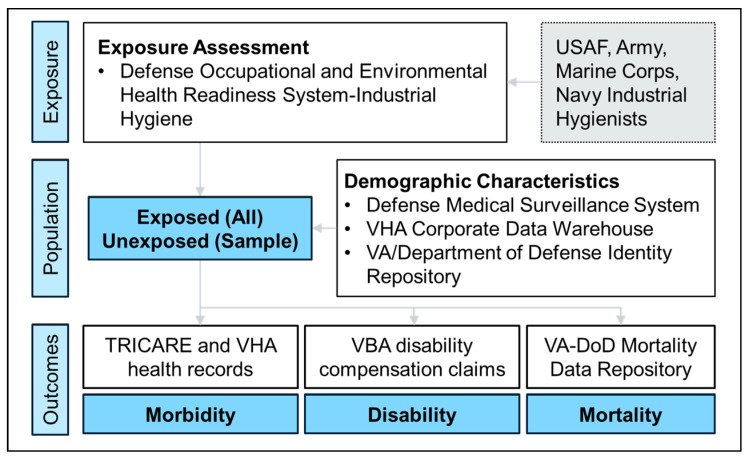
Flow diagram illustrating LIFE Study data sources. Jet fuel-exposed occupational codes were identified using the Department of Defense (DoD) Defense Occupational and Environmental Health Readiness System-Industrial Hygiene (DOEHRS-IH) and subject matter expertise from industrial hygienists in each of the services. The study population includes all Veterans occupationally exposed to jet fuel and a random sample of unexposed Veterans. The study roster is drawn from the DoD Defense Medical Surveillance System (DMSS) with supplemental demographic information drawn from the Veterans Health Administration (VHA) Corporate Data Warehouse (CDW) and the Veterans Affairs (VA)/DoD Identity Repository. The study roster is linked to TRICARE and VHA health records to assess morbidity outcomes, Veterans Benefits Administration (VBA) disability compensation claims to assess disability outcomes, and the VA-DoD Mortality Data Repository to assess mortality outcomes. Details on data elements from each source are available in Table S1. USAF, United States Air Force.

Figure 2 illustrates the linkages between the administrative data sources contributing to the study. A detailed breakdown of the specific data elements provided by each source can be found in Table S1.

#### 2.3.1. Defense Medical Surveillance System

**Figure 3:**
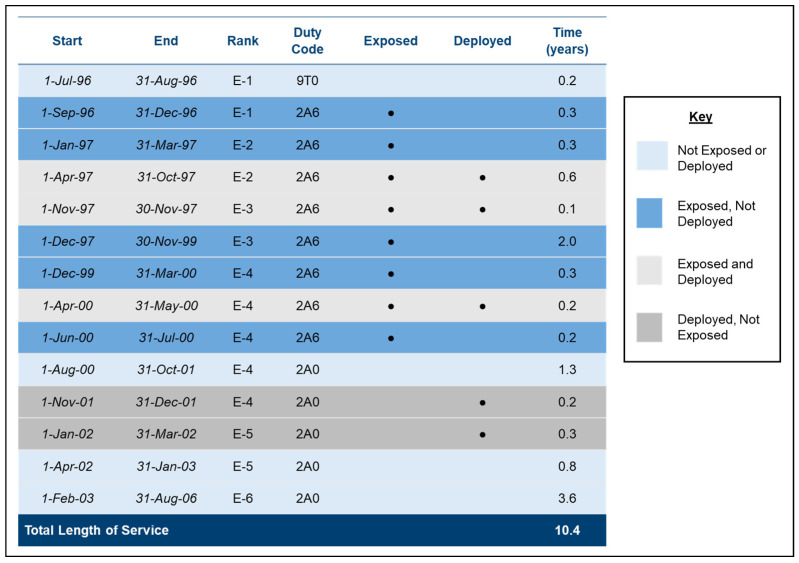
A hypothetical example of a jet fuel-exposed Air Force Veteran’s service history. Each line (or career segment) begins with a change in occupation, rank, or deployment status. The example Veteran had a total length of service of 10.4 years. Over the course of their Air Force career, they were promoted from Enlisted-1 (E1) to Enlisted-6 (E6). They spent 4 years in a jet fuel-exposed occupation (2A6, Aerospace Propulsion) and 6.4 years in unexposed occupations (9T0, Basic Enlisted Airman and 2A0, Avionics Test Station). The Veteran deployed three times, for a total of 1.4 years and spent 0.9 years concurrently deployed and in a fuel-exposed occupation. All these segments are included in the data for analysis.

The Defense Medical Surveillance System (DMSS) is the central repository for medical surveillance data for the US military. DMSS contains service member demographics (e.g., service member date of birth, race, and ethnicity), longitudinal service data from join date to separation (e.g., occupations, ranks, deployments, and duty locations) (Figure 3), and records of TRICARE insurance claims related to healthcare encounters for eligible individuals. It is maintained by the DoD Defense Health Agency (DHA) Armed Forces Health Surveillance Division (AFHSD).

Healthcare data in DMSS include administrative claims records from TRICARE, the military healthcare program for active-duty service members and eligible Veterans. TRICARE claims include International Classification of Diseases, 9th and 10th Revisions, Clinical Modification (ICD-9-CM, ICD-10-CM) diagnoses codes from all inpatient (since 1990) and outpatient (since 1996) direct care (military facility) and purchased care (private sector) encounters. It also includes pharmacy records since 2014 for medications covered by the TRICARE pharmacy benefit. TRICARE encounters that occurred during service are included for all service members and after separation from service for Veteran TRICARE enrollees only. Since 2008, records from encounters that occurred during deployment are contained in the Theater Medical Data Store (TMDS), maintained by the DoD Program Executive Office, Defense Healthcare Management Systems, and accessible via DMSS. Medical waivers submitted by service members with pre-existing conditions at the time of their enlistment are also available from DMSS. A full list of the data elements requested from DMSS for this study is listed in Table S1. TRICARE claims and pharmacy data will be refreshed every three to five years.

#### 2.3.2. Veterans Health Administration Corporate Data Warehouse

The Veterans Health Administration (VHA) Corporate Data Warehouse (CDW) contains records from Veterans’ healthcare encounters at the Veterans Health Administration. Eligibility for VHA services is determined based on military service characteristics, service-connected disability, and financial need. Enrolled Veterans access care through VA medical facilities or community care providers. Medical records stored in the VHA CDW include clinical data from inpatient and outpatient encounters, ICD-9-CM and ICD-10-CM diagnosis codes, pharmacy data, and health screening questionnaires. Table S1 lists variables extracted from the VHA CDW. These data will be refreshed every three to five years.

#### 2.3.3. VA-DoD Mortality Data Repository

The VA-DoD Mortality Data Repository is a comprehensive database of service member and Veteran mortality, which draws from the National Center for Health Statistics’ National Death Index and from DoD and VA administrative data sources. The VA-DoD Mortality Data Repository contains information from mortality reports for service members and Veterans who died on or after 1 January 1979, in any of the 50 US states, Washington, DC, Puerto Rico, and US territories. Date and cause of death for deceased members of the cohort were extracted from the VA-DoD Mortality Data Repository. Table S1 lists variables requested from the VA-DoD Mortality Data Repository. These data will be refreshed every five to ten years.

#### 2.3.4. Veterans Benefits Administration Enterprise Data Warehouse

The Veterans Benefits Administration (VBA) Enterprise Data Warehouse (EDW) is the centralized repository for all disability compensation claims filed by Veterans. Claims data in the VBA EDW are primarily populated by the Veterans Benefits Management System, the core platform for managing claims throughout their lifecycle, from initial submission and evidence gathering to adjudication and final decision notification. Medical conditions associated with disability claims are classified using a diagnostic coding system based on 38 Code of Federal Regulations Part 4, the Schedule for Rating Disabilities. This system categorizes conditions by disease type and organ system, serving as the foundation for evaluating the degree of disability and assigning compensation ratings. Each diagnostic code corresponds to specific criteria for determining the severity of a condition and its impact on earning capacity. The statutory authority underpinning this system, 38 USC. § 1155, ensures uniformity in disability evaluations across all claims. Information about claims, claimed conditions, and overall combined service-connected disability of Veterans submitting claims was requested from the VBA EDW. VBA generated flags indicating exposure-related considerations and special flags for fuel-related claims identified using keyword searches in rating decisions, diagnostic text, and claim attributes (e.g., “fuel,” “petroleum,” “jet fuel,” “benzene,” “hydrocarbons”) were also requested. Conditions unlikely to be related to fuel exposure (e.g., injuries) were excluded from the request. Table S1 lists variables requested from the VBA EDW. These data will be refreshed annually.

#### 2.3.5. Defense Occupational and Environmental Health Readiness System—Industrial Hygiene

The Defense Occupational and Environmental Health Readiness System-Industrial Hygiene (DOEHRS-IH) is a database maintained by the DHA Defense Health Services Systems to manage occupational and environmental exposure data related to operational risk management. DOEHRS-IH contains data related to standardized military occupational tasks (“processes”), associated potential health hazards (“process hazards”), and workplaces where these tasks are performed. It contains exposure monitoring data from personal breathing zone and area air samples related to specific process hazards (e.g., JP-8) collected from workplaces at military installations. DOEHRS-IH’s purpose is to integrate environmental health surveillance, occupational personnel exposure, workplace monitoring, and work practice observations to identify, evaluate, and control occupational and environmental health stressors [32]. DOEHRS-IH does not track exposures for individual service members; however, the occupations and ranks of service members routinely engaged in specific processes or present during workplace monitoring are recorded. DOEHRS-IH exposure monitoring data, including the occupation of personnel present at the time of measurement, and jet fuel hazard-associated process frequencies were requested from industrial hygienists in each of the services. Specific DOEHRS-IH data elements requested are provided in Table S1.

#### 2.3.6. VA/DoD Identity Repository (VADIR)

The VA/DoD Identity Repository (VADIR) is a system for sharing personnel data between DoD and VA, and the VHA CDW. VADIR and the VHA CDW were used as supplemental sources of demographic and service data when information was missing from DMSS. Table S1 lists variables extracted from VADIR. These data will be refreshed annually.

### 2.4. Exposure Assessment

Occupations likely to involve jet fuel exposure, hereafter referred to as jet fuel-exposed occupations, were identified by industrial hygienists from each of the services based on their association with processes and workplaces monitored for jet fuel hazards. The assessment integrated multiple types of data from DOEHRS-IH, including air samples, exposure characterizations, and risk assessments. Industrial hygienists also applied their subject matter expertise to identify non-exposed occupations incidentally appearing with jet fuel-related processes in DOEHRS-IH, and exposed occupations not appearing in the data. Common air- and ground-based, jet fuel-exposed occupations are provided in Supplementary Tables S2–S5. Veterans who worked in any of the jet fuel-exposed occupations at any time during their service were classified as jet fuel-exposed. Exposure duration was calculated for all jet fuel-exposed Veterans by summing years spent in any jet fuel-exposed occupations during their military careers (Figure 3).

Additional comparisons will be conducted by incorporating granular data on occupation-specific exposures. One approach will use similar exposure groups, aggregating service-specific occupations based on comparable work environments and performance of analogous fuel-related processes. Another approach will estimate individual cumulative exposure scores (CESs) combining individual time in exposed occupations with each occupations’ relative exposure intensity. For each exposed occupation, relative exposure intensity will be estimated using personal breathing zone air samples and other process-specific exposure data, along with reported frequencies of process occurrence. Time-weighted average (TWA) exposure levels will be multiplied by the frequency of each process, then averaged across all relevant processes associated with the occupation. For each individual participant, a CES will be calculated by multiplying the total time spent in each exposed occupation by that occupation’s relative exposure intensity. Individual CES values will be categorized as low, intermediate, or high exposure based on natural groupings within the study population

### 2.5. Data Linkage

The study roster obtained from DMSS was linked to TRICARE, TMDS, VHA CDW, VBA EDW, VA-DOD Mortality Data Repository, and VADIR using unique personal identifiers to obtain covariate and outcome information. The complete roster was compared against each database, and all matching records were extracted. Specific data elements used for the LIFE Study are detailed in Table S1. DMSS was considered the gold standard for demographic and service history data. Demographics (date of birth, sex, or race/ethnicity) were incomplete for 1.8% (25,004) of Veterans in the DMSS study roster. After supplementation with data from VADIR (first alternative) or the VHA CDW (second alternative), demographic information was incomplete for 0.8% of the study roster.

### 2.6. Outcome Assessment and Follow-Up

Various health outcomes will be investigated, with an emphasis on chronic, long-term outcomes. The initial focus for analyses will include outcomes previously identified as having slight evidence to suggest an association with jet fuel exposure in a previous systematic literature review. These include neurological, cognitive, behavioral, respiratory, and neoplastic outcomes [31]. Health outcomes will be assessed at multiple endpoints to develop a complete picture of Veterans’ long-term health. Table 1 provides a list of body systems and conditions that will be assessed in this study.

#### 2.6.1. Morbidity

The risk of acute, recurring, and aggravated disease following jet fuel exposure will be assessed using healthcare encounter diagnosis codes and pharmacy records from TRICARE, TMDS, and VHA. These outcomes will be measured using established ICD-9-CM and ICD-10-CM case definitions, such as those developed by AFHSD [33], whenever possible. Since utilization of TRICARE and VHA services varies among Veterans, to enhance internal validity, primary analyses of morbidity outcomes will include only Veterans actively engaged with the healthcare system (defined as at least one VHA encounter after separation or at least one TRICARE encounter at 90 days or more after separation). Sensitivity analyses will be employed to assess the generalizability of findings to Veterans who are not seeking care through TRICARE and VHA. A summary of LIFE Study Veterans eligible for these morbidity analyses, by fuel exposure status, is shown in Table 2.

For time-to-event analyses, person-time will be calculated for each member of the cohort. The start of follow-up will depend on the specific outcome and will continue up to one of three endpoints: date of the first encounter with a diagnosis of the outcome, date of death (from the VA-DoD Mortality Repository), or loss to follow-up. Individuals who have not experienced the outcome of interest and have no death record will be considered lost to follow-up on the earlier of two dates: (1) one year following their last VHA or TRICARE encounter, presuming that they are no longer receiving healthcare from VHA or TRICARE [34], or (2) the last date included in the DMSS dataset.

#### 2.6.2. Mortality

All-cause and cause-specific mortality outcomes will be examined using death records from the VA-DoD Mortality Data Repository. Analyses will use ICD-9 and ICD-10 codes for cause of death. Veterans who died from causes with uncertain or implausible connection to fuel exposure (e.g., operations of war and sequelae) will be excluded from mortality analyses. Excluded ICD codes are provided in Table S6. For time-to-event analyses, each Veteran’s follow-up period will begin at their separation from service and continue until date of death (from the VA-DoD Mortality Repository), or the last date included in the VA-DoD Mortality Data Repository.

#### 2.6.3. Disability

VA disability compensation claims will be used to assess disability associated with chronic disease following jet fuel exposure in the population of Veterans accessing VBA benefits. Disability analyses will be limited to the subset of Veterans with at least one submitted disability claim (for any condition) in the VBA EDW (Table 2). Disability compensation claim analyses may include the rate of claimed conditions, the proportion granted, and the disability rating percentage of granted claims. Claimed conditions deemed implausibly related to fuel exposure (e.g., musculoskeletal injuries) will be excluded from analyses. Since disability compensation claims alone cannot be used to determine prevalence of the outcomes of interest, these data will be considered supplementary to morbidity and mortality outcomes and can be used to elucidate the disability associated with chronic disease in the subset of the LIFE Study cohort that has accessed VBA benefits.

#### 2.6.4. Covariates

All analyses will consider demographic and military service-related covariates. Demographic covariates include race/ethnicity, sex, and age. Veterans’ attained age, calculated from date of birth, differs based on the requirements of specific analyses (e.g., age at the start of follow-up or at the time an outcome is diagnosed). Service-related covariates include service branch, deployment dates and locations, rank, and length of service.

Analyses may include smoking and alcohol dependence as indicated in VHA encounter and TRICARE claims data and health screening questionnaires. Smoking was identified using ICD-9-CM and ICD-10-CM diagnosis codes and free-text questionnaire responses [35,36]. Alcohol dependence was identified using ICD-9-CM and ICD-10-CM diagnosis codes only [37]. The ICD-9-CM and ICD-10-CM diagnosis codes used to identify smoking and alcohol dependence are provided in Table S7.

Covariates will be evaluated as both potential confounders and effect modifiers of the relationship between jet fuel exposure and health outcomes. Differences in the risk of health outcomes within and across sub-groups will be considered, including by deployment history, rank, service branch, race/ethnicity, and sex.

### 2.7. Statistical Analyses

Primary analyses of morbidity and mortality will utilize Cox proportional hazards models to assess time-to-event outcomes, adjusting for demographic and service-related covariates. Individual models will consider relevant characteristics of each outcome, such as latency period and recurrence. The duration of time spent in jet fuel-exposed occupations, the effect of CES (incorporating duration, intensity, and frequency of exposure, as described above) will also be analyzed to explore potential dose–response relationships.

Primary analyses of disability claims will utilize logistic regression models, adjusting for demographic and service-related covariates. Sensitivity analyses will consider the effect of VA policy changes which could contribute to changes in claim submission and approval rates over time, especially changes in presumptive conditions. Of particular importance to any claims-based analysis is the passage of the “Sergeant First Class Heath Robinson Honoring our Promise to Address Comprehensive Toxics Act of 2022” (PACT Act), which expanded VA health care and benefits for Veterans who deployed to certain conflicts and may have been exposed to toxic substances. Claims-based sensitivity analyses may consider confounding or effect modification based on PACT Act eligibility status of individual claims and by pre/post-PACT era.

Statistical approaches will be modified as necessary for individual analyses. Analysis-specific exclusion criteria may be used to ensure adequate data to assess the association between exposure and outcome exist among included individuals. Other statistical methods will be considered for specific outcomes of interest. For example, Poisson regression may be used to model the effect of jet fuel exposure on recurrent respiratory infections. Detailed methods, including additional exclusion criteria if used, will be documented for all analyses.

Interaction model analyses will attempt to identify effect modification by demographic and service-history covariates. Sensitivity analyses will also be used to assess the robustness of primary findings, including the potential impact of selection bias, exposure misclassification, and outcome case definitions. Veterans missing demographic information after linking to multiple databases will be excluded from all primary analyses; however, supplementary analyses may include these Veterans, using multiple imputation of missing variables. Veterans with significant gaps in their service history will also be excluded from analyses if their exposure status during the gap cannot be plausibly estimated.

#### Statistical Power

The large sample size of the LIFE Study provides substantial statistical power to detect clinically meaningful associations for most outcomes of interest, especially those with moderate-to-high prevalence in the cohort. For rare outcomes, the representativeness of the dataset supports exploratory analyses, although statistical power may be more limited. Given the large number of health outcomes to be assessed (Table 1) and the multiple population subsets (Table 2), conducting an over-arching power analysis for all outcomes is not feasible. However, power calculations will be performed at the outset of analyses for individual outcomes, as appropriate. All analyses will report confidence intervals to convey the precision of effect estimates, and results for rare outcomes will be interpreted with appropriate caution.

### 2.8. Ethical Considerations

The primary purpose of this operational investigation is to inform and improve services for Veterans by providing actionable information to VA and oversight entities. The LIFE Study protocol was reviewed by the Air Force Research Laboratory (AFRL) Institutional Review Board (IRB) and determined not to meet the definition of “research”, as defined by 32 CFR 219.102 [38].

Since the LIFE Study involves secondary analyses of pre-existing administrative records, Veterans in the cohort will not be contacted for additional information, or for consent to participate. Data sharing agreements are in place to ensure the secure transfer and handling of information between DoD and VA, with strict safeguards to protect the confidentiality of Veterans’ data. All analyses will be conducted in compliance with applicable federal privacy laws and VA data security policies. No identifiable personal information will be disclosed or published. All publications and presentations will adhere to VA’s publication standards and data suppression policies.

## 3. Results

Demographic and service history characteristics of the LIFE Study cohort are presented in Table 3. The cohort comprises 1,351,407 US Veterans, with 50.2% having at least one jet fuel-exposed occupation in their service history. Navy Veterans form the largest group (34.4%). Almost two-thirds (62.8%) of the cohort deployed at least once during their military career, and the vast majority (93.7%) held enlisted ranks at the time of their separation from service. On average, LIFE Study Veterans served for 6.8 years (standard deviation = 5.0 years), with 89.1% separating from service by age 35. As of 31 December 2023, the cohort’s age range spans from 19 to 83, with over 99% under 56 years old. The cohort is predominantly male (83.5%) and non-Hispanic white (61.2%).

Males make up a greater proportion of Veterans with any jet fuel-exposed occupation (85.8%) than unexposed Veterans (81.1%). Veterans with any jet fuel-exposed occupation were more likely to have deployed at least once (66.5% vs. 59.2% in unexposed) and less likely to have been an officer at their time of separation (1.9% vs. 6.9% in unexposed). Documented smoking history was more prevalent among Veterans with any jet fuel-exposed occupation (40.3%) than unexposed Veterans (36.3%), while alcohol dependence was similar in both groups (16.8% vs. 15.3%).

## 4. Discussion

The LIFE Study is a retrospective cohort that will leverage pre-existing administrative data to investigate the health effects of jet fuel exposure among Veterans, as determined by their military occupational history. Eligible Veterans with probable occupational exposure are assessed with a random sample of unexposed Veterans. Comprehensive data on military service, TRICARE claims and VHA health encounters, disability compensation claims, and mortality will be retrieved from DoD and VA databases to assess a wide range of health outcomes. Analytical priorities for this study will be determined based on the concerns of affected Veterans and other stakeholders, as well as signals observed in prior research.

The LIFE Study is the largest cohort to date to investigate the long-term health effects of occupational exposure to jet fuels in the military, consisting of over 1.3 million Veterans. Previous large cohorts in the United States [39,40] and Australia [41] have ranged in size from 14,457 to 893,941 members of the military, and none specifically evaluated the health of Veterans. The sample size of the current study provides unique statistical power to examine a wide range of health outcomes potentially linked to military jet fuel exposure, including rare conditions. Leveraging an administrative cohort eliminates the need for participation and follow-up over time, reducing the risk of selection bias. Moreover, the retrospective design will enable this study to address a significant gap in existing literature: assessment of long-term and chronic outcomes which emerge or persist after exposure has ended [31].

The demographics of the LIFE study cohort are consistent with the demographics of US Veterans who have served over the past 30 years. Sixty-one percent of the LIFE Study cohort is White, non-Hispanic, compared to 65.1% of post-9/11 Veterans and 76.7% of Veterans from all eras [42]. Likewise, the proportion of female Veterans in the LIFE Study population (16.5%) is similar to Gulf War and post-9/11 Veterans overall (14.6% and 16.8%, respectively) [42].

The military characteristics of the LIFE Study cohort, conversely, are distinct from members of the Millenium Cohort Study or Million Veterans Program with similar periods of service, and from the Veteran population overall [43,44]. First, Navy Veterans are overrepresented in the LIFE Study cohort. While they make up about one quarter of active-duty personnel in 2023 and 21.1% of Gulf Era Veterans in the Million Veterans Program [44,45], they represent the largest group in the LIFE Study (34.4%). Second, Veterans who were enlisted at the time of their separation are also overrepresented in the LIFE Study compared to current service members (over 93%, compared to 81.6% of active-duty personnel in 2023) [45]. One factor that may be contributing to these differences is the LIFE Study’s inclusion of all Veterans with any jet fuel-exposed occupation and a random sample of Veterans with no jet fuel-exposed code. Occupations identifying fuel-exposed military occupations were identified by industrial hygienists in each of the services, relying on workplace surveillance data from DOEHRS-IH. Overall, these codes identified enlisted occupations rather than officer occupations, leading to a high proportion of enlisted Veterans in the cohort. Likewise, a high proportion of Navy Veterans had a fuel-exposed occupation in their service history, contributing to their overrepresentation. Another factor that may affect the military characteristics of the LIFE Study cohort is its exclusion of Veterans who ever served in the National Guard or Reserve. While the active duty Army is only about 30% larger than the active duty Navy or Air Force, among service members who transferred from the active component to reserve component between 2010 and 2016, more than twice as many separated from the Army than from any other service (about 75,000) [45,46]. Air Force transfers were a distant second (approximately 30,000). Because Army service members are more likely than other service members to serve in the National Guard or Reserve, they may be less likely to be included in the LIFE Study cohort. While it is not representative of all Veterans, the LIFE Study cohort is representative of jet fuel-exposed Veterans who served exclusively in the active-duty military.

By analyzing longitudinal service histories, the study will examine health outcomes associated with varying durations of occupational jet fuel exposure. This approach will facilitate the observation of cumulative effects and dose–response relationships, a substantial gap in the current literature [31]. This retrospective cohort design is especially effective for studying chronic diseases of all body systems and conditions with long latency periods, offering a resource- and time-efficient approach to measuring long-term health outcomes.

The use of administrative data will also mitigate recall bias in exposure assessment. DMSS duty rosters provide detailed occupational and deployment histories for all Veterans, regardless of health status. While it is possible that Veterans concerned about environmental hazards may be more likely to seek healthcare or to file disability claims after separation, this potential bias will be limited by including only Veterans with documented healthcare encounters, pharmacy records, or disability compensation claims in primary analyses.

A strength of the LIFE Study is its inclusion of a random sample of unexposed Veterans as a reference group. Both exposed and unexposed Veterans likely had similar physical health and fitness levels prior to and during service, experienced comparable environmental exposures other than jet fuels had similar access to healthcare during their service. The inclusion of unexposed Veterans will mitigate the “healthy soldier effect” commonly seen in comparisons between active-duty personnel or Veterans and the general population [47], including previous studies of occupational fuel exposure [40,48].

While the LIFE Study improves upon previous research methods, some remaining limitations are anticipated. First, this study uses military occupation as a surrogate for jet fuel exposure. While this is a common approach when direct measurement is not available for all members of the cohort [11,39,40,49,50,51,52], it may result in misclassification of exposure status. This misclassification could arise from inaccurate characterization of occupations as exposed or unexposed. It could also result from variations in exposure intensity among service members with the same job title, depending on their specific duties in different locations or services. To limit exposure misclassification, industrial hygienists used measurement data from DOEHRS-IH to classify jet fuel-exposed occupations. Detailed occupational histories were used to measure cumulative time spent in jet fuel-exposed occupations as a proxy for exposure duration. Since exposed and unexposed occupations are equally likely to be misclassified, occupational exposure misclassification is believed to be non-differential. This could possibly bias results toward an underestimate of the health effects of jet fuel exposure.

Unmeasured jet fuel (or related chemical) exposures outside of military service, including non-occupational and post-separation exposures are another potential source of exposure misclassification. While non-occupational exposures are likely non-differential, post-separation occupational exposures may be more prevalent among Veterans who worked in fuel-related military occupations, potentially leading to an underestimation of the total exposure duration. While this could result in some overestimation of exposure effects, it is unlikely to produce entirely false associations.

There are also limitations related to the use of administrative data for health outcome ascertainment in the LIFE Study. Although this method is cost-saving and efficient, a significant number of post-separation healthcare encounters may not be captured in either VHA or TRICARE records because many Veterans receive civilian healthcare covered by private insurance, Medicaid, or Medicare. In 2019–2021, 16.6% of Veterans aged 25–64 were enrolled in TRICARE, 11.8% had VA healthcare only, and 66.5% had public or private insurance, with or without VA healthcare [53]. Health care visits and diagnoses occurring outside of TRICARE and VHA due to dual insurance enrollment could lead to a non-differential under- or overestimation of disease prevalence, reduced statistical power for rarer health outcomes, and inaccurate event timing. Furthermore, Veterans who exclusively use VHA and TRICARE may differ systematically from those who primarily receive care elsewhere, risking the introduction of selection bias [54,55,56]. In one study, VA researchers found that, compared with non-users, VA healthcare users had greater risk of several chronic medical conditions—in particular, chronic fatigue, arthritis, sleep apnea, diabetes, hypertension, migraines, irritable bowel disease, significant hearing loss, and sinusitis—after adjusting for demographic and military service characteristics. Bias related to health and socio-demographic differences in VA healthcare users and Veteran non-users may be inherent in working with VA encounters data [57].

To reduce the effect of these limitations, the LIFE Study will use validated case definitions, including AFHSD’s surveillance case definitions [33], to ensure consistent outcome ascertainment, and tailor analyses to maximize internal validity by selecting appropriate analysis populations, modeling approaches, and index dates. Sensitivity analyses will further assess the impact of missing healthcare data on findings and the generalizability of findings to all exposed Veterans.

Similarly, analysis of disabilities in Veterans exposed to jet fuels relies on submitted disability compensation claims. In fiscal year 2024, approximately 41% of Veterans who served between 1990 and 2024 received disability compensation from VBA [58]. However, this group may not fully represent the entire population of jet fuel-exposed Veterans. Several factors, such as awareness of potential health effects, access to resources, and individual decisions to file claims, can vary widely, influencing the likelihood of submitting a compensation claim and the outcome of adjudication.

The interpretation of claims data must consider potential sources of bias. Veterans who file claims may be more likely to perceive or experience adverse health outcomes, creating selection bias. Conversely, some Veterans with legitimate health concerns may not file claims due to lack of awareness, stigma, or systemic barriers to accessing benefits. The adjudication process may also introduce variability, as compensation decisions depend on the strength and clarity of evidence provided, as well as the regulatory criteria applied. To enhance the internal validity of disability claims analyses, only Veterans who have submitted at least one claim will be included in primary analyses focused on disability. This approach ensures a more consistent basis for comparing outcomes.

While granted claims are definitive indicators of recognized disability, denied claims do not necessarily indicate the absence of a disability. Research has shown that Veterans with denied claims often experience significant challenges, such as fewer financial resources, reduced social support, and similar or worse physical and psychosocial functioning compared to those with granted claims [59,60,61]. Given these complexities, both granted and denied claims will be included in supplementary analyses to understand the relationship between jet fuel exposure and disability outcomes.

Bias in mortality ascertainment is unlikely in the LIFE Study, as the mortality data originate from the National Death Index, the most complete source of death information in the United States. However, since 99% of the study population is under 56 at the end of 2023, extended follow-up periods will be required to adequately capture many disease-related mortality outcomes, such as heart disease and cancer. Premature analyses of these outcomes could possibly underestimate the impact of jet fuel exposure on mortality. To address this, the LIFE Study will refresh datasets to assess health outcomes, including mortality, at regular intervals.

Finally, analyses cannot be adjusted for confounding variables that are not captured by the administrative datasets, such as family history of disease. This limitation is inherent in all secondary analyses of administrative data. To reduce the impact of this limitation, a random, representative, reference population was selected for all analyses, and adjustment will be made for all measured confounders.

Analyses of health outcomes will be prioritized based on stakeholder interest and previous studies. Initially, outcomes in body systems that the VA’s systematic literature reviews have identified as likely to be targeted by jet fuel exposure (i.e., the nervous and sensory systems, respiratory system, cancer, and mental health) will be assessed [31]. Further, specific outcomes noted in previously published cohort studies can also be evaluated. For instance, the LIFE Study will build upon Knave et al.’s 1976 finding that chronic depression and anxiety was associated with fuel exposure in Swedish jet engine factory workers, by replicating their findings in a larger cohort with clearly defined exposures and outcomes [15]. Likewise, D’Este et al. observed higher rates of cancer-related mortality in Australian fuel tank de-seal/re-seal participants [41]. The LIFE Study offers the opportunity not only to validate those findings in a larger, more diverse cohort, but also to broaden the scope by investigating how exposure to jet fuel affects cancer morbidity and associated disabilities.

## 5. Conclusions

The LIFE Study is the largest study to date to investigate the long-term health effects of occupational jet fuel exposure. Extensive data available from DoD and VA will allow for assessments of a wide variety of conditions across all body systems. A key strength of this study is its ability to analyze fuel exposures accumulated over entire careers, providing a more complete picture of lifetime risk. Regular updates of healthcare encounters, disability claims, and mortality data will ensure that findings capture long-term outcomes, including disease-related deaths, as they become more prevalent in the cohort over time.

Jet fuel is a common exposure among military service members in the US and internationally. Ultimately, this study will have broad implications for policies related to healthcare and disability benefits for Veterans, as well as for the development of safeguards to limit future occupational exposures during military service. By leveraging pre-existing administrative data, this study also demonstrates a cost-effective and innovative approach to improving both care delivery and policy development. The LIFE Study’s approach sets a precedent for investigating chronic and long-latency health effects following occupational exposures in Veteran populations.

## Figures and Tables

**Table 1:** Body systems and health conditions that will be examined in the LIFE Study.

Body System or Health Condition
Cancer ^1^ Cardiovascular
Dermal
Developmental
Digestive
Endocrine
Hematologic
Hepatic
Immune Mental health ^1^
Metabolic
Musculoskeletal and connective tissue
Nervous and sensory ^1^
Renal
Reproductive
Respiratory ^1^

^1^ Systems and conditions with slight epidemiologic evidence to suggest an adverse effect of occupational jet fuel exposure in previous studies [31].

**Table 2:** LIFE Cohort Veterans with post-separation healthcare encounters or disability benefits claims.

	Full Cohort		Jet Fuel Exposure ^1^			
			Exposed		Unexposed	
	N	%	N	%	N	%
Total ^2^	1,340,859	100	673,217	50.2	667,642	49.8
Post-separation health care encounters ^3^						
TRICARE (at least one, more than 90 days after separation)	38,810	2.9	19,464	2.9	19,346	2.9
VHA (at least one, ever)	878,487	65.5	446,732	66.4	431,755	64.7
Either TRICARE or VHA encounters	888,134	66.2	451,452	67.1	436,682	65.4
Veterans disability benefits claims						
Ever filed a claim	993,706	74.1	504,697	75.0	489,009	73.2

^1^ Jet fuel exposure status is determined by the presence of any jet fuel-exposed military occupation in a Veteran’s service history. For a complete list of jet fuel-exposed occupations, see Supplementary Tables S2–S5. ^2^ Excludes veterans missing any demographic covariate. ^3^ Overall, 83 Veterans (0.01%) missing information on both TRICARE and VHA claims.

**Table 3:** Demographic and Service History Characteristics of the LIFE Study Cohort.

	Full Cohort		Jet Fuel Exposure ^1^			
			Exposed		Unexposed	
	N	% ^2^	N	% ^2^	N	% ^2^
Total	1,351,407	100	677,804	50.2	673,603	49.8
Service branch						
Army	406,679	30.1	198,267	29.3	208,412	30.9
Air Force	216,918	16.1	106,891	15.8	110,027	16.3
Marines	263,090	19.5	133,569	19.7	129,521	19.2
Navy	464,720	34.4	239,077	35.3	225,643	33.5
Deployment						
Deployed	849,331	62.8	450,856	66.5	398,475	59.2
Not deployed	502,076	37.2	226,948	33.5	275,128	40.8
Rank at separation						
Enlisted	1,266,534	93.7	651,702	96.2	614,832	91.3
Warrant officer ^3^	7143	0.5	6020	0.9	1123	0.2
Officer	77,674	5.8	20,068	3.0	57,606	8.6
Length of service, years (mean, SD)	6.8	5.0	7.0	5.2	6.5	4.7
Age at separation						
18–25	712,531	52.7	361,096	53.3	351,435	52.2
26–35	492,037	36.4	237,092	35.0	254,945	37.8
36–45	132,535	9.8	72,758	10.7	59,777	8.9
46–55	13,768	1.0	6767	1.0	7001	1.0
56 and older	523	0.0	89	0.0	434	0.1
Sex						
Male	1,128,263	83.5	581,778	85.8	546,485	81.1
Female	223,143	16.5	96,026	14.2	127,117	18.9
Race/ethnicity						
Non-Hispanic Asian or Pacific Islander	49,283	3.6	23,955	3.5	25,328	3.8
Non-Hispanic Black	209,316	15.5	108,818	16.1	100,498	14.9
Hispanic	185,984	13.8	96,692	14.3	89,292	13.3
Non-Hispanic Native American or Alaska Native	21,662	1.6	11,535	1.7	10,127	1.5
Non-Hispanic Other race	47,957	3.5	23,624	3.5	24,333	3.6
Non-Hispanic White	826,664	61.2	408,595	60.3	418,069	62.1
History of smoking ^4^						
Yes	517,945	38.3	273,442	40.3	244,503	36.3
No	833,462	61.7	404,362	59.7	429,100	63.7
History of alcohol dependence ^5^						
Yes	216,883	16.0	113,642	16.8	103,241	15.3
No	1,134,524	84.0	564,162	83.2	570,362	84.7

^1^ Jet fuel exposure status is determined by the presence of any jet fuel-exposed military occupation in a Veteran’s service history. For a complete list of jet fuel-exposed occupations, see Supplementary Tables S2–S5. ^2^ Percentages may not sum to 100% due to missing values. Overall, 0.8% of the LIFE Study cohort is missing any demographic covariate. Missing sex, N = 1 (0.0%); missing race and ethnicity, N = 10,541 (0.8%); missing rank at join date, N = 56 (0.0%); missing age at separation, N = 13 (0.0%). ^3^ Warrant officer is a category of ranks above senior enlisted ranks but below officer grade O-1 in the US Army, Navy, and Marine Corps. ^4^ History of smoking is identified using ICD-9-CM and ICD-10-CM diagnosis codes and free-text questionnaire responses, validated by McGinnis et al., 2011 [35] and McGinnis et al., 2022 [36], documented in either TRICARE or VHA health records. The list of codes is provided in Table S7. Veterans with “no” history of tobacco use may have a history of tobacco use that is not documented in either TRICARE or VHA health records. ^5^ History of alcohol dependence is identified using ICD-9-CM and ICD-10-CM diagnosis codes, developed Bergman et al., 2020 [37], documented in either TRICARE or VHA health records. The full list of codes is provided in Table S7. Veterans with “no” history of alcohol dependence may have a history of alcohol dependence that is not documented in either TRICARE or VHA health records.

## Data Availability

The data used in this study are provided by the US Department of Veterans Affairs (VA) and the US Department of Defense (DoD) by permission. Data will be shared upon reasonable request to the corresponding author with permission of VA and DoD.

## Supplementary Materials

The following supporting information can be downloaded at https://www.mdpi.com/article/10.3390/ijerph22091337/s1; Table S1. Government-owned Administrative Data Sources Providing Data to the LIFE Study; Table S2. US Army Military Occupational Specialty (MOS) Codes for Jet Fuel-Exposed Occupations; Table S3. US Air Force Specialty Codes (AFSC) for Jet Fuel-Exposed Occupations; Table S4. US Navy Occupational Coding Systems for Enlisted Service Members and Officers with Jet Fuel-Exposed Occupations; Table S5. US Marine Corps Military Occupational Specialty (MOS) Codes for Jet Fuel-Exposed Occupations; Table S6. ICD-10 Cause-of-Death Codes Excluded from Analyses; Table S7. ICD-10-CM Codes for Nicotine and Alcohol Dependence.

## Abbreviations

The following abbreviations are used in this manuscript:

VA	Department of Veterans Affairs
DoD	Department of Defense
USAF	United States Air Force
LIFE	Long-term Impact of Fuel Exposure
VHA	Veterans Health Administration
CDW	Veterans Health Administration Corporate Data Warehouse
VBA	Veterans Benefits Administration
EDW	Veterans Benefits Administration Enterprise Data Warehouse
DMSS	Defense Medical Surveillance System
DHA	DoD Defense Health Agency
AFHSD	Armed Forces Health Surveillance Division
ICD-9-CM, ICD-10-CM	International Classification of Diseases, 9th and 10th Revisions, Clinical Modification
TMDS	Theater Medical Data Store
DOHERS-IH	Defense Occupational and Environmental Health Readiness System—Industrial Hygiene
TWA	Time Weighted Average
CES	Cumulative Exposure Score
VADIR	VA/DoD Identity Repository
AFRL	Air Force Research Laboratory
IRB	Institutional Review Board
