# Supplementary material for: The Long-Term Impact of Fuel Exposure (LIFE) Study: A Tri-Service Cohort of United States Veterans with Military Occupational Exposure to Jet Fuels
Source: Int J Environ Res Public Health. 2025 Aug 27;22(9):1337. doi: 10.3390/ijerph22091337 (PMC12470227; doi:10.3390/ijerph22091337)
Supplement: Supplementary file 1 [file ijerph-22-01337-s001.zip › ijerph-3794379-supplementary.pdf]

Supplementary Information

The Long-term Impact of Fuel Exposure (LIFE) Study: A Tri-Service Cohort of Veterans with Military Occupational Exposure to Jet Fuels

Elizabeth Heitz, MPH[1]
Nicholas Tilton, PhD[1]
Justin G. Bergeron, BVMS, MPH, DACVPM[1]
Gregory Wolff[2]
Jennifer A. Rusiecki, PhD[3]
Aaron I. Schneiderman, RN, MPH, PhD[4]
W. Scott Monks, MPAS, PA-C[5]
Christopher Edwards[6]
Gillon D. Marchetti, Esq.[7]
Terra D. Vincent-Hall, PhD, DABT[1]

Affiliations:

1 Exposure Science Program, Health Outcomes Military Exposures, Veterans Health Administration, Department of Veterans Affairs, Washington, DC 20420, USA

2 Epidemiology Consult Service, US Air Force School of Aerospace Medicine, Wright-Patterson AFB, OH 45433, USA

3 Department of Medicine, Uniformed Services University of the Health Sciences, Bethesda, MD 20814, USA

4 Epidemiology Program, Health Outcomes Military Exposures, Veterans Health Administration, Department of Veterans Affairs, Washington, DC 20420, USA

5 Directorate of Clinical Public Health and Epidemiology, Defense Centers for Public Health-Aberdeen, Aberdeen Proving Ground, MD 21010, USA

6 711th Human Performance Wing, US Air Force School of Aerospace Medicine, Wright-Patterson AFB, OH 45433, USA

7 Military Exposure Team, Compensation Service, Veterans Benefits Administration, Department of Veterans Affairs, Washington, DC 20420, USA

Contents

Table S1. Government-owned Administrative Data Sources Providing Data to the LIFE Study 1

Table S2. U.S. Army Military Occupational Specialty (MOS) Codes for Jet Fuel-Exposed Occupations 4

Table S3. U.S. Air Force Specialty Codes (AFSC) for Jet Fuel-Exposed Occupations 5

Table S4. U.S. Navy Occupational Coding Systems for Enlisted Service Members and Officers with Jet Fuel-Exposed Occupations 6

Table S5. U.S. Marine Corps Military Occupational Specialty (MOS) Codes for Jet Fuel-Exposed Occupations 8

Table S6. ICD-10 Cause-of-Death Codes Excluded from Analyses 9

Table S7. ICD-9-CM and ICD-10-CM Codes for Smoking and Alcohol Dependence 10

Table S1. Government-owned Administrative Data Sources Providing Data to the LIFE Study

Database	Initial retrieval date and planned refresh interval	Information	Variables
**U.S. Department of Defense-owned Systems**			
Defense Medical Surveillance System (DMSS)	Records through December 31, 2023 retrieved December 13, 2024.<br><br>Refresh of TRICARE claims planned every 3-5 years.	Cohort roster, demographics, and military service history	Military service history<br>• Join date<br>• Military occupation code (start and end dates)<br>• Rank (start and end dates)<br>• Deployment (start and end dates)<br>• Separation date<br>Demographic variables<br>• Sex<br>• Race<br>• Ethnicity<br>• Birthdate
		TRICARE medical claims	Inpatient encounters<br>• Admission date<br>• ICD-9/10-CM codes (up to 9 diagnostic positions)<br>• Health screening questionnaires (direct care only)<br>Outpatient encounters<br>• Visit date<br>• ICD-9/10-CM codes (up to 9 diagnostic positions)<br>• Health screening questionnaires (direct care only)<br>• Setting type<br>Pharmacy records
		Theater-based medical encounters (from the Theater Medical Data Store)	Inpatient encounters<br>• Admission date<br>• ICD-9/10-CM codes (up to 9 diagnostic positions)<br>Outpatient encounters<br>• Visit date<br>• ICD-9/10-CM codes (up to 9 diagnostic positions)
		Medical waivers	Conditions diagnosed prior to joining the service

Defense Occupational and Environmental Health Readiness System – Industrial Hygiene (DOEHRS-IH)	Records through December 31, 2023 retrieved January 5, 2024.<br><br>No data refresh planned.	Exposure monitoring data	Exposure monitoring data<br>• Personal breathing zone measurements<br>• Area air sample measurements<br>• Personnel present at time of measurement<br>Processes involving jet fuel<br>• Process frequencies
**U.S. Department of Veterans Affairs-owned systems**			
Veterans Affairs/ Department of Defense Identity Repository (VADIR)	Records through December 31, 2023 retrieved December 13, 2024.<br><br>Refresh planned annually.	Supplemental data on cohort demographics and military service history	Military service history<br>• Deployment start and end dates<br>• Deployment location<br>Demographic variables<br>• Sex<br>• Race<br>• Ethnicity<br>• Birthdate
VA-DoD Mortality Data Repository	Records not yet retrieved.<br><br>Refresh planned every 5-10 years.	Veteran vital status	Vital status<br>• Date of death<br>• Cause of death
Veterans Benefits Administration (VBA) Enterprise Data Warehouse (EDW)	Records through December 31, 2023 retrieved January 30, 2025.<br><br>Refresh planned annually.	Veterans disability compensation claims submitted	Disability compensation claims<br>• Unique claim submission identifiers<br>• Claimed medical conditions<br>• Diagnostic codes<br>• Decision type (e.g., granted, denied, deferred)<br>• Percentage of disability assigned to a service-connected condition<br>• Date of claim<br>• Exposure-related considerations flag (e.g., toxic exposure, fuel exposure indicators, or deployment to hazardous locations
Veterans Health Administration Corporate Data Warehouse (VHA CDW)	Records through December 31, 2023 retrieved December 24, 2024<br><br>Refresh planned every 3-5 years.	VHA healthcare encounters	Inpatient encounters<br>• Admission date<br>• ICD-9/10-CM codes (up to 9 diagnostic positions)<br>• Health screening questionnaires<br>Outpatient encounters<br>• Visit date<br>• ICD-9/10-CM codes (up to 9 diagnostic positions)

			• Health screening questionnaires<br>• Setting type<br>Pharmacy records
		Supplemental cohort demographics	Demographic variables<br>• Sex<br>• Race<br>• Ethnicity<br>• Birthdate

Table S2. U.S. Army Military Occupational Specialty (MOS) Codes for Jet Fuel-Exposed Occupations

MOS Code	Career Field
12N	Horizontal Construction Engineer
15A through 15Z	Aviation Branch, Enlisted
19D	Cavalry Scout
74D	Chemical, Biological, Radiological and Nuclear (CBRN) Specialist
88M	Motor Transport Officer
88N	Transportation Management Coordinator
89B	Ammunition Specialist
91B	Wheeled Vehicle Mechanic
92A	Quartermaster Officer / Automated Logistical Specialist, Enlisted
92F	Petroleum Supply Specialist
92L	Petroleum Laboratory Specialist
92Y	Unit Supply Specialist
150A	Air Traffic and Air Space Management Technician
150U	Unmanned Aircraft Systems Operation Technician
151A	Aviation Maintenance Technician (Nonrated)
152B through 152H	Warrant Officer Pilot
153A, -B, -D, -L, -M	Warrant Officer Pilot
154C, -E, -F	Warrant Officer Pilot
155E, -F, -G	Warrant Officer Pilot

Table S3. U.S. Air Force Specialty Codes (AFSC) for Jet Fuel-Exposed Occupations

AFSC	Career Field
1A1[a]	Aircrew Operations
1A2	Aircraft Loadmaster
1A9	Special Missions Aviator
21A	Aircraft Maintenance Officer
21MxA	Conventional Munitions Officer
2A3	Tactical Aircraft Maintenance
2A5	Airlift/Special Mission Aircraft Maintenance
2A6	Aerospace Propulsion
2A7	Aircraft Metals Technology Specialist
2A8	Mobility Air Forces Integrated Communication / Navigation / Mission Systems
2F0	Fuels
2T3	Mission Generation Vehicular Equipment Maintenance
2W1	Aircraft Armament Systems
3E4	Water and Fuel Systems Maintenance Specialist
3E7	Fire Protection

[a]AFSC 1A1x8 (Executive Mission Aviator) was excluded from analyses.

Table S4. U.S. Navy Occupational Coding Systems for Enlisted Service Members and Officers with Jet Fuel-Exposed Occupations

Service Members	
**Enlisted Rating**	**Career Field**
PR	Aircrew Survival Equipmentman
AW	Naval Aircrewman
AN	Airman
AB	Aviation Boatswain's Mate
AE	Aviation Electrician's Mate
AT	Aviation Electronics Technician
AD	Aviation Machinist's Mate
AO	Aviation Ordnanceman
AM	Aviation Structural Mechanics
AME	Aviation Structural Mechanic, Safety Equipment
AS	Aviation Support Equipment Technician
BM	Boatswain's Mate
DC	Damage Controlman
EN	Engineman
EO	Equipment Operator
FT	Fire Control Technician (Submarine)
FC	Fire Controlman
GSE	Gas Turbine Systems Technician, Electrical
GSM	Gas Turbine Systems Technician, Mechanical
GM	Gunner's Mate
HT	Hull Maintenance Technician
MR	Machinery Repairman
MM	Machinist's Mate
MN	Mineman
MT	Missile Technician

Table S4 *(continued)*. U.S. Navy Occupational Coding Systems for Enlisted Service Members and Officers with Jet Fuel-Exposed Occupations

Officers	
**Officer Code**	**Career Field**
111	Unrestricted Line (URL) Officer qualified as a Surface Warfare Officer
112	URL Officer qualified as a Submarine Warfare Officer.
113	URL Officer qualified as a Special Warfare Officer (SEAL)
114	URL Officer qualified as an Explosive Ordnance Disposal (EOD) Warfare Officer
130	URL Officer previously qualified Naval Aviator or Naval Flight Officer
131	URL Officer qualified for duty involving piloting as a Naval Aviator
144	Restricted Line (RL) Officer - Engineering Duty Officer qualified as a Ship Engineering specialist
152	RL Officer - Aerospace Engineering Duty Officer, Maintenance (AMDO and AMO)
310	Supply Corps Officer
510	Civil Engineer Corps (i.e., Seabee) Officer
611	Limited Duty Officer - Deck (Surface)
613	Limited Duty Officer - Engineering/Repair (Surface)
616	Limited Duty Officer - Ordnance (Surface)
623	Limited Duty Officer - Engineering/Repair (Submarine)
631	Limited Duty Officer - Deck (Aviation)
633	Limited Duty Officer - Maintenance (Aviation)
636	Limited Duty Officer - Ordnance (Aviation)
711	Warrant Officer - Boatswain (Surface)
713	Warrant Officer - Engineering Technician (Surface)
716	Warrant Officer - Ordnance Technician (Surface)
731	Warrant Officer - Boatswain (Aviation)
734	Warrant Officer - Engineering Technician (Aviation)
736	Warrant Officer - Ordnance Technician (Aviation)

Table S5. U.S. Marine Corps Military Occupational Specialty (MOS) Codes for Jet Fuel-Exposed Occupations

MOS Code	Career Field
11	Utilities
13[a]	Engineer, Construction, Facilities and Equipment
35	Motor Transport
60	Aircraft Maintenance
61	Aircraft Maintenance (Rotary-Wing)
62	Aircraft Maintenance (Fixed-Wing)
70[b]	Airfield Services
75	Pilots/Naval Flight Officers
7313	Helicopter Specialist, AZ-1Z/UH-1Y
8972	Aircrew Trainee
9972	Aircrew Trainee
9973	Fixed-Wing Transport Aircraft Specialist, KC-130J
9976	Helicopter Specialist, AH-1Z/UH-1Y

[a]MOS Codes 1330 (Facilities Management Officer) and 1361 (Engineer Assistant) were excluded from analyses.
[b]MOS Codes 7041 (Aviation Operations Specialist) and 7077 (Weapons and Tactics Instructor – Aviation Ground Support) were excluded from analyses.

Table S6. ICD-10 Cause-of-Death Codes Excluded from Analyses

ICD-10 Code[a]	Description
Q00 – Q99	Congenital malformations, deformations, and chromosomal abnormalities
R00 – R99	Symptoms, signs, and abnormal clinical and laboratory findings, not elsewhere classified
Y40 – Y84, Y88.0 – Y88.3	Complications of medical and surgical care and sequelae
Event of Undetermined Intent	
Y10 – Y19	Poisoning
Y20	Hanging, strangulation, and suffocation
Y21	Drowning and submersion
Y22 – Y24	Firearm discharge
Y26	Exposure to smoke, fire, and flames
Y30	Falling, jumping, or pushed from a high place
Y25, Y27 – Y29, Y31 – Y34, Y87.2, Y89.9	Other and unspecified means and sequelae
Legal Intervention	
Y35.0	Legal intervention involving firearm discharge
Y35.5	Legal execution
Y35.1 – Y35.4, Y35.6 – Y35.7, Y89.0	Other and unspecified means and sequelae
Y36, Y89.1	Operations of war and sequelae

[a]ICD-10 codes identifying causes with uncertain or implausible connection to fuel exposure. ICD-10 codes will be cross-walked to ICD-9 codes.

Table S7. ICD-9-CM and ICD-10-CM Codes for Smoking and Alcohol Dependence

Smoking[a]	
**ICD-10-CM Code**	**Description**
F17	Nicotine dependence
O99.33x	Tobacco use (smoking) during pregnancy, childbirth, and the puerperium
T65.2x	Toxic effect of nicotine
Z71.6	Tobacco use counseling
Z72.0	Tobacco use NOS
Z87.891	History of tobacco dependence
**ICD-9-CM Code**	**Description**
305.1	Tobacco use disorder
V15.82	Personal history of tobacco use
Alcohol Dependence[b]	
**ICD-10-CM Code**	**Description**
F10.1x	Alcohol abuse
F10.2x	Alcohol dependence
**ICD-9-CM Code**	**Description**
291.0	Alcohol withdrawal delirium
291.81	Alcohol withdrawal
303.00	Acute alcoholic intoxication in alcoholism, unspecified
303.03	Acute alcoholic intoxication in alcoholism, in remission
303.90	Other and unspecified alcohol dependence, unspecified
303.93	Other and unspecified alcohol dependence, in remission
305.00	Alcohol abuse, unspecified
305.01	Alcohol abuse, continuous
305.02	Alcohol abuse, episodic

[a]ICD-10-CM code list validated by McGinnis et al, 2022. ICD-9-CM codes were cross-walked from ICD-10-CM codes.
[b]ICD-10-CM code list developed Bergman et al, 2020. ICD-9-CM codes were cross-walked from ICD-10-CM codes.
